# Glottic configuration in wind instrument players

**DOI:** 10.1016/S1808-8694(15)30033-1

**Published:** 2015-10-19

**Authors:** Claudia Alessandra Eckley

**Affiliations:** a PhD from the FCMSCSP; Fellow in Professional Voice - Thomas Jefferson University - Philadelphia, Assistant Professor Santa Casa de Misericórdia de São Paulo.

**Keywords:** glottis, wind instrument, dysphonia

## Abstract

**Introduction:**

Knowledge of occupational voice disorders has gained increased importance as more individuals rely on their voices for their work. Wind instrument players are a specific group of individuals that present intensive use of the vocal tract associated with blowing their instrument. Interestingly, only a small number of reports focus on the laryngeal function of such professionals.

**Aim:**

The current study evaluated the laryngeal and vocal tract movement of wind instrument players.

**Materials and Methods:**

Ten adult wind instrument players were studied with flexible videolaryngoscopy while playing their instrument, in order to observe the movements of the glottis, the vocal tract and the base of the tongue.

**Results:**

In all the participants it was observed that musical tones were played with adducted vocal folds and that the greater the technical difficulty reported by the player, the more it was associated with increased lateral tension in the larynx, as well as constriction in the vocal tract.

**Conclusions:**

The larynx controls the airflow that will reach the mouthpiece of the instrument, directly interfering in blowing. Therefore, wind instrument players should also be considered professional voice users.

## INTRODUCTION

Knowledge about professional-voice related health problems is increasingly important as more and more persons use their voices as working tools^1^, ^2^, ^3^, ^4^. Different studies have identified some occupational groups as more prone to develop voice alterations, such as teachers, singers, actors, telemarketing operators and sales people^2^, ^4^, ^5^. Musicians who play wind instruments represent a very specific group of individuals who make intense use of their vocal tract in their professional activities. Curiously, very little or nothing has been reported about the larynx direct participation in this profession. In part, we owe this to the historic notion that vocal chords would not have a direct action in producing the instrument sound, the effort would be made only by the respiratory and oromandibular muscles; and the larynx should be open only to allow air flow^3^, ^4^, ^5^, ^6^. The goal of this study is to evaluate wind instrument musicians’ larynx and their vocal tract behaviors in order to better understand the real contribution of these anatomical structures in these professionals.

## MATERIALS AND METHODS

10 wind instrument musicians were studied, 6 males and 4 females, with average age of 42.5 years. Inclusion factors were: to play a wind instrument as a major means of making a living, not to make any other type of professional voice use, not to have previous history of laryngeal or pulmonary diseases. All individuals who fit this criteria consented in participating in the study after being properly informed about the objectives and proposed diagnostic techniques.

The subjects were questioned about their general health and vocal complaints related to voice use and playing the wind instrument. Following that, the participants underwent videolaryngoscopy with flexible optic fiber without topical anesthesia. After documenting the anatomy of their larynx and pharynx the individual would play his/her wind instrument. We asked them to play a song that would be considered easy and another one considered technically challenging. The exams were recorded in video and analyzed later on.

The parameters observed were: position of the vocal chords and the vestibular folds during sound production; antero-posterior and lateral larynx diameter during sound production; tongue base and pharynx position and behavior while they were blowing the instrument; presence or absence of vibrato and its probable location in the vocal tract.

## RESULTS

The main complaints found were throat secretion (8/10) and dysphonia after intense use of the instrument (5/10). In all participants we could see that the musical tones were produced during the adduction of the vocal chords. The airflow and blowing control was apparently related to the changes between glottic constriction and opening. Vibrato was present as glottic opening and closing rhythmic movements. We also noticed that when they played a more difficult song there was a greater glottic (side-to-side constriction) and supra-glottic tension. Other laryngeal signs found were arytenoid and interarytenoid edema and hyperemia in 8 of the 10 participants, of moderate intensity in 5, and mild in 3. No vocal chord lesions were seen.

## DISCUSSION AND CONCLUSION

The scarce existing literature on the health problems of wind instrument musicians is concentrated on disorders of diaphragm control or embouchure^5^, ^8^. By embouchure we mean the use of the oromandibular muscles, most specially the orbicularis oris, in order to blow through the instrument mouth piece^5^. Regarding the later, there are some very interesting reports about dental and denture problems, highlighting the importance of these alterations in blowing quality^5^, ^6^. Other health problems related to the professional use of a wind instrument are of neurological nature – focal oromandibular dystonias – in this group of patients^5^, ^8^. A paper showing an increase in intraocular pressure in the individuals who play wind instruments also suggest a great importance of the valvular capacity in order to control airflow based on the increase in air pressure within vocal tract and high respiratory tract^9^. The concept that in order to produce proper air flow for the wind instrument the larynx should be relaxed and the vocal chords abducted has led us to negligence for many years, that being the possible larynx participation in the production and control of sound by these individuals. Notwithstanding, a common problem found in this group was of dysphonia after intense use of the wind instrument, even when they did not “utter a word”. With the current knowledge about the important glottic participation in airflow regulation, it would be only logical to suppose that this anatomical structure has active participation in blowing the instrument. This has been systematically proved by our study. The only similar study found in the literature was one from Mukai, 1989^7^, who also studied wind instrument players’ larynges through nasofibroscope, observing the direct glottic participation in airflow control in blowing the instrument. This author stated that the embouchure was very important as air flow receiver from the respiratory tract and regulated by the glottis. We concluded that the glottis has active participation in wind instruments sound production and that glottic configuration alterations may interfere in the final musical sound. This knowledge suggests the need to include wind instrument musicians in the group of the so called voice professionals, and study them as to their specific vocal demands (type of wind instrument, working environment, other parallel vocal activities, etc.).
